# Hyperferritinemia screening to aid identification and differentiation of patients with hyperinflammatory disorders

**DOI:** 10.21203/rs.3.rs-4523502/v1

**Published:** 2024-06-25

**Authors:** Hallie A. Carol, Adam S. Mayer, Michael S. Zhang, Vinh Dang, Jemy Varghese, Zachary Martinez, Corinne Schneider, Joy (Elizabeth) Baker, Paul Tsoukas, Edward M. Behrens, Randy Q. Cron, Caroline Diorio, Lauren A. Henderson, Grant Schulert, Pui Lee, Kate F. Kernan, Scott W. Canna

**Affiliations:** Division of Pediatric Rheumatology, The Children’s Hospital of Philadelphia; Division of Pediatric Rheumatology, The Children’s Hospital of Philadelphia; Division of Pediatric Allergy/Immunology, University of Pittsburgh School of Medicine, UPMC Children’s Hospital of Pittsburgh; Division of Pediatric Rheumatology, The Children’s Hospital of Philadelphia; Division of Pediatric Rheumatology, The Children’s Hospital of Philadelphia; Division of Pediatric Oncology, The Children’s Hospital of Philadelphia; RK Mellon Institute for Pediatric Research & Pediatric Rheumatology, University of Pittsburgh School of Medicine, UPMC Children’s Hospital of Pittsburgh; Division of Pediatric Rheumatology, Cincinnati Children’s Hospital Medical Center; Division of Pediatric Rheumatology, Hospital for Sick Children,Toronto, ON; Division of Pediatric Rheumatology, The Children’s Hospital of Philadelphia; Division of Pediatric Rheumatology, The University of Alabama at Birmingham; Division of Pediatric Oncology, The Children’s Hospital of Philadelphia; Division of Immunology, Boston Children’s Hospital; Division of Pediatric Rheumatology, Cincinnati Children’s Hospital Medical Center; Division of Immunology, Boston Children’s Hospital; Department of Critical Care Medicine, University of Pittsburgh School of Medicine, UPMC Children’s Hospital of Pittsburgh; Division of Pediatric Rheumatology, The Children’s Hospital of Philadelphia

**Keywords:** Hyperferritinemia, Hyperinflammatory disorders, Hemophagocytic Lymphohistiocytosis (HLH), Macrophage Activation Syndrome (MAS), Interleukin-18

## Abstract

High ferritin is an important and sensitive biomarker for hemophagocytic lymphohistiocytosis (HLH), a diverse and deadly group of cytokine storm syndromes. Early action to prevent immunopathology in HLH often includes empiric immunomodulation, which can complicate etiologic work-up and prevent collection of early/pre-treatment research samples. To address this, we instituted an alert system where serum ferritin > 1000ng/mL triggered real-time chart review, assessment of whether the value reflected “inflammatory hyperferritnemia (IHF)”, and biobanking of remnant samples from consenting IHF patients. We extracted relevant clinical data; periodically measured serum total IL-18, IL-18 binding protein (IL-18BP), and CXCL9; retrospectively classified patients by etiology into infectious, rheumatic, or immune dysregulation; and subjected a subgroup of samples to a 96-analyte biomarker screen. 180 patients were identified, 30.5% of which had IHF. Maximum ferritin levels were significantly higher in patients with IHF than with either hemoglobinopathy or transplant, and highly elevated total IL-18 levels were distinctive to patients with Stills Disease and/or Macrophage Activation Syndrome (MAS). Multi-analyte analysis showed elevation in proteins associated with cytotoxic lymphocytes in all IHF samples when compared to healthy controls and depression of proteins such as ANGPT1 and VEGFR2 in samples from hyperferritinemic sepsis patients relative to non-sepsis controls. This single-center, real-time IFH screen proved feasible and efficient, validated prior observations about the specificity of IL-18, enabled early sample collection from a complex population, suggested a unique vascular biomarker signature in hyperferritinemic sepsis, and expanded our understanding of IHF heterogeneity.

## Introduction

Hyperferritinemia is a critical component in the diagnosis of a spectrum of cytokine storm syndromes (CSS) that includes macrophage activation syndrome (MAS) and hemophagocytic lymphohistiocytosis (HLH). MAS occurs in rheumatic diseases, most commonly systemic juvenile idiopathic arthritis or adult-onset Stills disease (sJIA and AOSD, respectively; hereafter, collectively called Stills Disease), but also complicates systemic lupus erythematosus, Kawasaki disease, juvenile dermatomyositis, and autoinflammatory diseases(1). HLH most famously arises due to genetic causes (primary HLH), including severe genetic impairment of cytotoxic function (familial HLH, FHL). It occurs much more commonly secondary to underlying infection or malignancy. In addition to hyperferritinemia, hallmark features of HLH/MAS include cytopenias, liver injury, hepato- and/or splenomegaly, disseminated intravascular coagulation, and histologic hemophagocytosis(2). HLH/MAS can occur in nearly any inflammatory context, and treatment and prognosis can vary dramatically depending on specific infectious/inflammatory triggers, genetic susceptibilities, and underlying diseases with mortality ranging from 5 to 40%(3). The underlying immunopathology makes patients at risk of organ failure and death(4). Therefore, early and appropriate interventions are critical for minimizing morbidity and mortality.

Despite the importance of early identification, we currently lack specific diagnostic biomarkers. Diagnostic and classification criteria for MAS and HLH(5–7) rely on routine laboratory tests (e.g. ferritin, cell counts, liver transaminases, fibrinogen, triglycerides) that are individually non-specific and on specialized testing (NK function, soluble IL-2R, CD107a mobilization, bone marrow biopsy) that does not perform much better and take a prolonged time to result(8). In practice, individual test results do not capture dynamic changes in lab values and were not developed for early recognition. Hyperferritinemia can be driven by inflammation and/or immune dysregulation, but is also observed in malignancy, infection, and iron overload states(9). Nevertheless, hyperferritenemia is associated with mortality across all hospitalized children(10).

Elevation of IFNγ-induced biomarkers like CXCL9 and IL-18 binding protein (IL-18BP) are associated with disease activity in both MAS and HLH(11–13). In retrospective studies, dramatic elevation of total interleukin-18 (IL-18), with detectable free IL-18, has been associated with MAS. Though promising, these specialized tests are not obtained on a routine clinical basis.

To improve recognition of patients with CSS, we instituted a novel hyperferritinemia alert system. By screening all patients with substantially elevated ferritin values, we hoped to identify the landscape of hyperferritinemic patients, collect the earliest samples for research purposes, and test these samples for HLH/MAS-related biomarkers.

## Materials and Methods

### Informed consent

was obtained from all patients or their legal guardians, and all study procedures were approved by the Institutional Review Boards of the University of Pittsburgh (STUDY20010099) or Children’s Hospital of Philadelphia (CHOP, IRB #18–014863). Samples obtained via hyperferritinemic screen were obtained in the period of June 1st, 2017 to June 30th, 2019, with clinical data abstracted from the electronic health record of the UPMC Children’s Hospital of Pittsburgh. Samples for Olink analysis (see below) were obtained on the same protocols but not necessarily via hyperferritinemic screen. Olink analysis samples were supplemented with Cytokine Release Syndrome (CRS) samples obtained from CHOP.

### Hyperferritinemia Screening

**Figure 1:**
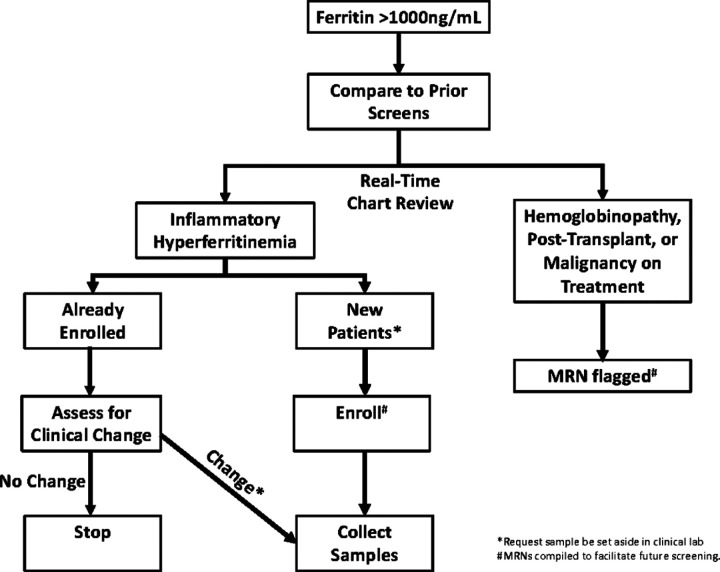
Steps in the hyperferritinemia screening protocol.

Despite many criteria using ferritin cutoffs from 500–700ng/ml, to improve specificity a cutoff of 1000ng/ml was used in this study. Serum ferritin levels measured at the UPMC Children’s Hospital of Pittsburgh clinical laboratory greater than 1000ng/ml were compiled into an email alert to study PI (SC) twice per week (Fig. 1). The medical records of patients generating alerts were screened and adjudicated by consensus of study team members (SC, MZ, KK). Patients were categorized into five groups based on real-time chart review: inflammatory hyperferritinemia (IHF), hemoglobinopathy (e.g. sickle cell disease, beta-thalassemia, etc.), malignancy undergoing treatment, post-transplant (solid-organ or hematopoietic), and “other”. Only patients adjudicated as IHF were eligible for study enrollment and sample collection; patients in the “malignancy” and “post-transplant” groups were excluded due to the complexity of their contributors to hyperferritinemia. Alerts adjudicated as IHF deriving from new (i.e. unenrolled) patients generated a request to the clinical laboratory that remnant sera (kept at 4°C) be set moved to a designated area at −20°C in the clinical laboratory. Sera were kept at 4°C for less than 72 hours prior to freezing. Such patients and/or their guardians were then approached for participation in a natural history study. If enrolled, further chart review was performed and patients were further sub-categorized by consensus of the study team into rheumatologic, infection-induced, and immune dysregulation (i.e. IHF not due to rheumatologic or infectious causes) groups (Table S1). Frozen sera from unenrolled patients were discarded after 3 months.

Subsequent alerts from enrolled patients with IHF were assessed for whether they represented a clinical change based on real-time chart review by study team members. Alerts associated with a clinical change generated a request that remnant serum be set aside. Medical record numbers, enrollment status (enrolled, declined, unable to contact) patients, and IHF adjudication (yes/no) were continuously compiled into a separate file available only to study team members to facilitate rapid screening of future alerts.

Subsequently, a hyperferritinemic (ferritin > 1000ng/mL) screen was performed at Cincinnati Children’s Hospital Medical Center (CCHMC) from January 3, 2021 to June 30, 2021. Informed consent was obtained from all patients or their legal guardians, and all study procedures were approved by the Institutional Review Boards of CCHMC (IRB2018–2408).

### Chart review and sample testing

At the conclusion of two years’ data collection, laboratory components of HLH/MAS diagnostic criteria (platelet count, triglycerides, fibrinogen, AST) and C-reactive protein (CRP) drawn contemporaneously with the elevated ferritin level were abstracted from the medical record of all patients in the IHF group. If multiple clinical laboratory assessments were performed within 24 hours of a ferritin assessment, the most elevated (for ferritin, CRP, triglycerides, or AST) or depressed (for platelet count and fibrinogen) values were abstracted. To reduce confounding by frequency of sampling, we examined the most extreme laboratory values (occurring during periods of hyperferritinemia) from distinct patients and compared them across subgroups. Bio-banked serum samples were tested for IL-18, IL-18BP, and CXCL9 levels by Luminex bead-based immunoassay as previously described(11). Bridging controls were used to assure consistency between sample runs.

Hyperferritinemic (> 1000 ng/mL) samples from the screen, supplemented with IHF samples biobanked on the same protocols, low grade CRS samples (CRS, grade 0–1, ferritin range 35–3000 ng/ml), and healthy controls were analyzed by Olink Target 96 Immuno-Oncology panel (Olink Proteomics, Uppsala, Sweden). IHF had clinically defined diagnosis (e.g. SJIA-MAS, fHLH, sepsis, etc) as determined by treating clinicians per chart review. Analyte results were excluded from the analysis if > 75% of the samples had Normalized Protein Expression (NPX) values below the limit of detection (Table S2).

### Statistical analysis

Statistical analyses were performed as described in the figure legends using GraphPad Prism v10 (San Diego, CA, USA). The Kruskal-Wallis test was performed to assess for the presence of a statistically significant difference in laboratory values between multiple patient groups. Dunn’s multiple comparison test was then used to compare between individual groups or subgroups. An adjusted p-value < 0.05 was considered statistically significant. Standardized Principal Component Analysis (PCA) with components based on parallel analysis, and associated component loading coefficients, were obtained using Prism v10.

## Results

### Clinical diagnoses of patients with positive ferritin screens

**Figure 2:**
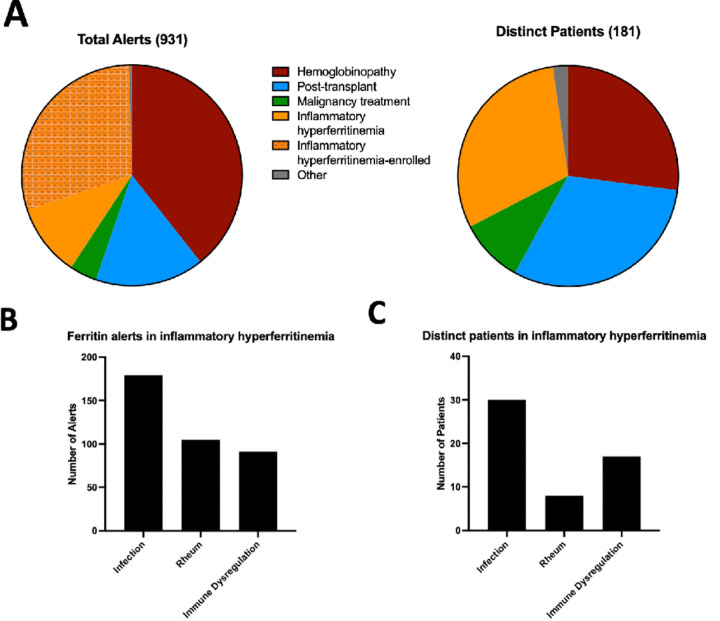
Distribution of total hyperferritinemia alerts and distinct patients triggering alerts by disease category (**A**). Bar graphs display the number of alerts per subgroup of inflammatory hyperferritinemia (**B**) and the number of distinct patients represented in each subgroup (**C**).

Over a two-year period, 931 alerts were received for elevated ferritin levels > 1000 ng/ml in 180 distinct patients. Patients in the hemoglobinopathy group generated the largest numbers of new alerts, followed by the post-transplant, IHF, malignancy and “other” groups (Fig. 2A). Patients termed “other” included GM2 synthase deficiency, aortic arch reconstruction, neonatal hemochromatosis, and myasthenia gravis.

Patients adjudicated as IHF generated 40.3% of total alerts from 55 distinct patients (30.5% of total patients). Alerts from patients previously enrolled during the study period comprised nearly 75% of the total alerts for the group. After sub-stratification of patients in the IHF group, we found that the infection subgroup represented both the largest number of distinct patients and the most alerts (Fig. 2B–C). 40% of patients in this subgroup had underlying sepsis, 17% had Epstein-Barr virus (EBV) infection, 7% had adenovirus and the remainder had a range of other infections including influenza and cystic fibrosis pneumonia (**Table S2**). Patients with rheumatologic hyperferritinemia generated the second most alerts, but these derived from only eight patients (14.5% of inflammatory hyperferritinemia patients), all of whom had Stills Disease. The remainder of the alerts were generated from a diverse “immune dysregulation” subgroup, which was comprised of patients with common variable immunodeficiency (CVID), trisomy 18, XIAP deficiency, Shimke Immuno-Osseous Dysplasia, and several undifferentiated immune dysregulation diseases characterized by necrotizing pancreatitis, acute multi-organ system failure, or immunodeficiency. In the CCHMC ferritin screen, 35 patients (56.7%) would be defined as IHF with 24 (32%) having Multisystem Inflammatory Syndrome in Children (MIS-C) or active COVID-19 and only 1 patient with sepsis (Table S3).

### Laboratory characteristics of screened patients

**Figure 3:**
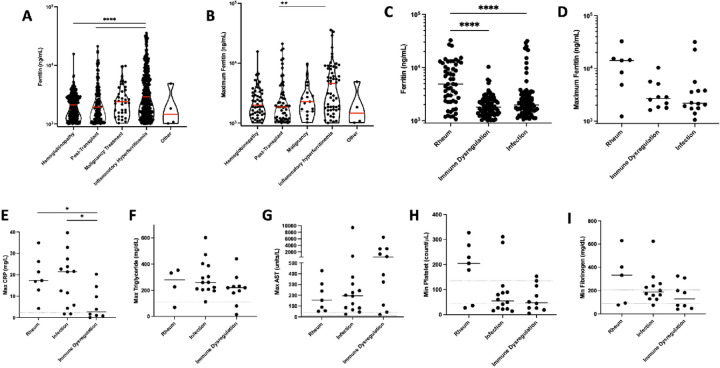
Distribution of ferritin values and maximum ferritin value per distinct patient by disease group for all alerts (**A-B**). Of enrolled patients, all and maximum ferritins by subgroup of inflammatory hyperferritinemia (**C-D**), and maximum values for other HLH-related laboratory criteria by subgroup of inflammatory hyperferritinemia (**E-I**). *p<0.05, **p<0.01, ***p<0.001, ****p<0.0001 Kruskal-Wallis with Dunn’s post-test; only comparisons with p<0.05 shown.

Examining the ferritin distribution, we found that the median ferritin value for IHF patients was significantly higher than both the hemoglobinopathy and transplant groups. We identified a similar pattern, albeit less statistically significant, in the distribution of maximum ferritin values (Fig. 3A–B).

Within the IHF group, patients with rheumatologic hyperferritinemia had a significantly higher mean ferritin compared to the infection and immune dysregulation subgroups (p < 0.0001 for both). This general trend continued, but did not reach significance, when only comparing individual patient maximum ferritin values (Fig. 3C–D). Assessing other markers of hyperinflammation across the subgroups of inflammatory hyperferritinemia, we found all three subgroups mounted varying levels of acute inflammatory or tissue damage responses. Both infection and rheumatologic groups showed significantly higher maximum CRP levels than the immune dysregulation group (P = 0.037, P = 0.047 respectively). There was no significant difference in maximum triglyceride, minimum platelet, or minimum fibrinogen levels between groups. Though not statistically significant, there was a trend toward higher AST elevation in the immune dysregulation group (Fig. 3E–I).

**Figure 4:**
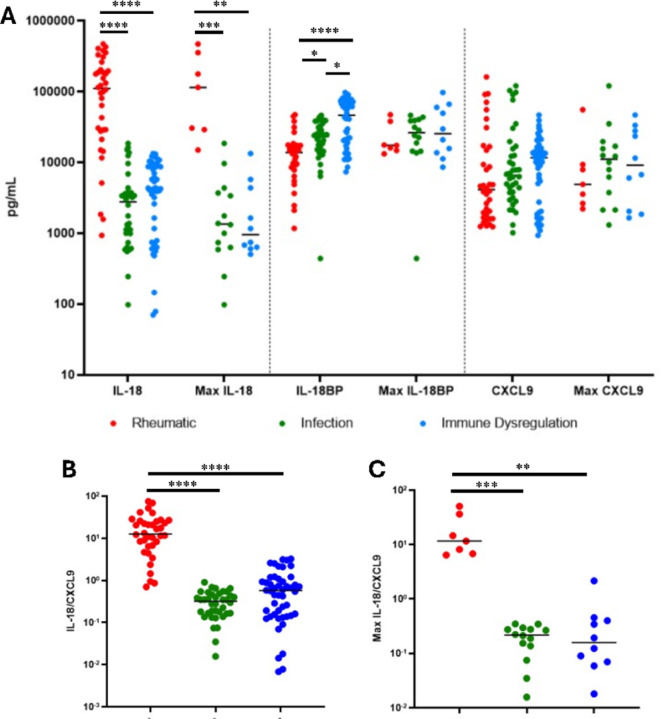
IL-18, IL-18BP and CXCL9 levels by subgroup of inflammatory hyperferritinemia (**A**). Patients with rheumatic disease had significantly higher IL-18 levels (P<0.0001 for both) and significantly lower IL-18BP levels (P<0.05, P<0.0001, respectively) compared to those with infection or immune dysregulation. Panel **B** shows the distribution of IL-18/CXCL9 ratios amongst the subgroups with rheumatologic patients having significantly higher ratios than those in the other two subgroups (P<0.0001). *p<0.05, **p<0.01, ***p<0.001, ****p<0.0001 Kruskal-Wallis with Dunn’s post-test; only comparisons with p<0.05 shown.

IHF biobanked samples were tested for specialized hyperinflammatory biomarkers, IL-18, IL-18BP and CXCL9. As expected, patients with rheumatologic hyperferritinemia (Stills Disease) had significantly higher total IL-18 (includes free IL-18 and inactive IL-18 bound to IL-18BP) values (both per sample and maximum) compared to the infection and immune dysregulation subgroups (Fig. 4A). Inversely, patients with rheumatologic hyperferritinemia had the lowest IL-18BP level of the three subgroups. CXCL9 levels did not significantly differ between IHF subgroups. Accordingly, IL-18/CXCL9 ratios were significantly higher in rheumatologic-associated hyperferritinemia compared to the other two subgroups. These ratios were not significantly different when compared between the infection and immune dysregulation subgroups (Fig. 4B). Prior experience suggested the utility of longitudinal assessment of traditional and specialized HLH biomarkers in disease activity monitoring(14, 15). In two selected patients in this cohort, similar longitudinal screening highlighted the utility of IL-18 as a biomarker of treatment efficacy and risk for relapse (Figure S1A & B, respectively).

### Multi-analyte analysis to identify candidate discriminators of IHF etiology

To determine novel biomarker candidates that could aid in the identification and stratification of IHF, we performed an Olink Target 96 Immuno-Oncology panel (chosen for most extensive coverage of targets of interest) on IHF biobanked samples from 86 subjects with Stills-MAS (n = 18), HLH (primary, n = 5; malignancy-associated, n = 5, and infection-associated, n = 8), sepsis (n = 25, with annotation of whether it was bacteria and/or viral), low-grade CRS (grades 0, n = 6: grade 1, n = 9), and healthy controls (n = 10) (Table S4). Primary HLH included deficiency of PRF1, STXBP2, and XIAP. All infection-associated HLH samples were due adenovirus or EBV and none required medications for blood pressure support.

**Figure 5:**
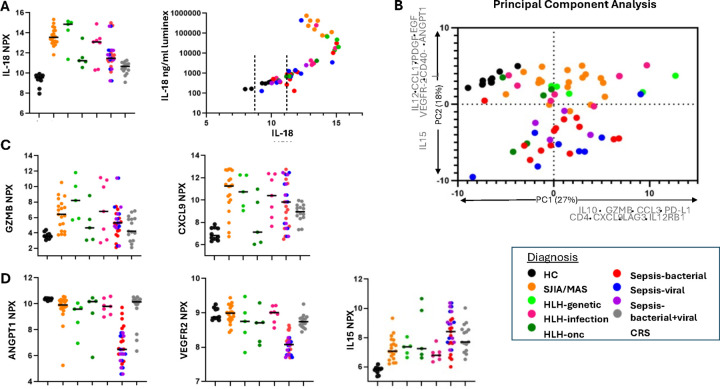
Biomarker screen using Olink. **A**. Left: Analysis of IL-18 NPX values does not show levels uniquely elevated in SJIA/MAS samples compared to other hyperferritenemic samples. Right: Correlation between IL-18 NPX values and IL-18 measured via Luminex as in Weiss et al. [9]. Dashed lines indicate IL-18 NPX values of the low-grade CRS samples for which Luminex IL-18 values were not available. **B**. Principal component analysis shows unsupervised clustering of analyte NPX values with PC1 accounting for 27% of the variability and PC2 accounting for 18%. The analytes with the highest absolute value contribution to PC loading are listed on their respective axes (full list of PC loadings in Supplemental Table 3). **C**. Both CXCL9 and Granzyme B contribute to PC1 and separate hyperferritinemic samples from healthy controls (all graphs contributing to PC1 in Supplemental Figure 3). **D**. ANGPT1, VEGFR2, and IL15 contribute to PC2. ANGPT1 and VEGFR2 are downregulated and IL15 is upregulated in hyperferritinemic septic samples compared to other hyperferritinemic samples and healthy controls (all graphs contributing to PC2 in Supplemental Figure 4). Abbreviations: PDGF= PDGF subunit B.

We first compared IL-18 values measured by bead-based assay with O-link NPX values. Unexpectedly, IL-18 NPX values from Stills-MAS samples were not significantly elevated over other disease groups (Fig. 5A). Correlation between bead-based IL-18 concentrations and NPX IL-18 values demonstrated a typical “hook effect” (a.k.a. prozone phenomenon). Whereas the O-link assay is run on samples without dilution, we diluted samples 25-fold for our bead-based assay because we previously made similar observations at low dilutions(11). IL-18 NPX values were therefore excluded from further analyses. Though no other orthogonally-measured values of other analytes tested were available for this assay, we found a similar hook effect for IL-18 and CXCL9 measured using the O-link Target 48 cytokine panel compared to Luminex (IL-18) and Legendplex (CXCL9) assays (**Figure S2**). We re-analyzed an aptamer-based study in Stills and related conditions(16) in which both aptamer (SomaLogic) and Luminex values were available for CXCL9 and IL-18BP (total IL-18 was not included in the aptamer assay). We did not observe a hook effect for CXCL9 or IL-18BP in the aptamer assay, but the range of CXCL9 values measured by Luminex-based assay in this study was far narrower than those measured by Legendplex panel, suggesting fewer very high CXCL9 concentrations in the samples from the aptamer study (**Figure S3**).

Nevertheless, we aimed to identify biomarkers capable of distinguishing IHF subtypes. After removing IL-18, we took an unsupervised, “diagnosis-agnostic” approach by principal component analysis (PCA). Healthy control samples clustered separately from most IHF samples (Fig. 5B). All other IHF samples were distributed similarly along Principal Component 1 (PC1). The analytes with the strongest contribution to PC1 included proteins conspicuously associated with T cell/NK cell activation (e.g. Lymphocyte activity, LAG3), cytotoxicity (Granzyme B), and/or IFNγ activity (CXCL9) (Fig. 5B, C, **Figure S4, Table S5**). By contrast, PC2 largely distinguished hyperferritinemic sepsis samples from the remainder of the cohort, including healthy controls. PC2 was less circumscribed, but included endothelial/vascular proteins and growth factors (e.g. ANGPT1, VEGFR2, PDGF) and proteins associated with antigen presenting cell activation (e.g. CD40L, IL-15) (Fig. 5B, D, **Figure S5, Table S5**). Low grade CRS samples were not consistently hyperferritinemic and, in a separate analysis, largely clustered with healthy controls (**Figure S6**)

## Discussion

Owing to its complexity, heterogeneity, severity, and rapid evolution, severe systemic inflammation poses both biologic and clinical challenges. This complexity is reflected by the terms used to describe it, including SIRS, sepsis, HLH, MAS, cytokine storm. Like most complex disorders, it results from the interplay of genetic susceptibility factors, background diseases, and inciting triggers, and these features all affect presentation and severity. Timely identification, proper diagnosis, and prompt context-specific management are integral to preventing immunopathology and clinical deterioration(8). Ferritin has consistently cited as a critical screening biomarker for HLH and related disorders(5–7), potentially improved by dividing by ESR(17). To aid in identifying and monitoring such patients, we implemented a novel hyperferritinemia alert system and ultimately screened 931 alerts from 180 patients over a two-year period. Overall, the process was feasible with minimal staff, clinically useful in identifying and tracking patients at risk for systemic immunopathology, and it significantly aided efforts to collect early research specimens. These features suggest feasibility and generalizability to other care settings.

Clinical ferritin testing served as the basis for this study, and we did not advertise the study or attempt to influence ferritin ordering practices. Non-inflammatory hyperferritinemia, largely from hemoglobinopathies like sickle cell anemia and post-transplant patients, made up the majority of both total alerts and individual patients. Inflammation may contribute to hyperferritinemia in these patients, including HLH, but we excluded these groups from further analysis given their frequent ferritin testing as part of iron panels and the major contribution of iron overload to their hyperferritinemia. In general, the distribution of diagnoses associated with IHF and its correlation with HLH vary substantially by cut-off value and institutional testing practices(18–20). In our cohort, patients with Stills Disease generated the second most alerts. Ferritin is used routinely for screening and monitoring in Stills, and these diagnoses are far more common than primary HLH. The high proportion of alerts from sepsis patients likely reflects a long-standing institutional interest in hyperferritinemic sepsis(10, 21). This is in contrast to the CCHMC where ferritin is only ordered when requested or as part of a clinical pathway leading to the CCHMC ferritin screen only identifying 1 patient with sepsis, while capturing a high proportion of MIS-C patients, as ferritin is part of a clinical pathway. However, it is important to note that while ferritin levels are more routinely obtained as part of clinical practice at UPMC Children’s Hospital, the case mix of sepsis patients is not abnormal. It may reasonably be expected that if ferritin levels were obtained universally, a similar case mix of IHF would be seen at other institutions. Institutional ferritin ordering practices will influence the patients identified and ultimately the samples that are able to be collected.

Overall, our findings reinforce the reality that the readily-available components of the HLH/MAS classification criteria(5–7) lack specificity for underlying/driving diagnoses. Rheumatologic (Stills) patients had significantly higher mean ferritin values and trends toward less coagulopathy (higher platelet and fibrinogen levels) than the other two subgroups, consistent with prior literature (22, 23), but these findings appeared too non-specific to be of significant clinical utility in distinguishing causes of CSS. Patients with “immune dysregulation” had lower maximum CRP levels, possibly reflecting differences in underlying inflammatory processes. As such, we measured total IL-18, IL-18BP, and CXCL9, so-grouped because their degree of elevation in these diseases requires a high dilution(11). Consistent with prior retrospective data(11, 24), and growing clinical experience, significantly elevated total IL-18 and a high IL-18/CXCL9 ratio distinguished hyperferritinemic Stills patients from the other two IHF subgroups(25). As CXCL9 did not significantly differ between groups, the utility of this ratio is largely driven by IL-18 levels.

The ferritin alert system also facilitated longitudinal assessment of patients with chronic hyperferritinemia, enabling an anecdotal assessment of the utility of biomarker trends. We found that intermittent assessment of IL-18 over a patient’s clinical course was a useful residual disease activity marker, with elevation showing active or imminent flares and improvement/normalization predicting durable response (or lack thereof) to treatment (**Figure S1**). Thus, IL-18 may be useful both diagnostically and in assessing minimal residual disease activity in Stills disease.

Multi-analyte panels typically use the same sample dilution for all analytes measured, rendering both antibody- and aptamer-based platforms susceptible to hidden hook effects, particularly at analyte levels above what assay developers might encounter in healthy and common disease controls. In a 96-plex “immuno-oncology” assay of 80 curated serum samples, our “positive control” analyte (IL-18) failed to distinguish Stills patients. Given prior experience(11), we suspected and subsequently identified a strong hook effect at high total IL-18 concentrations. We found similar hook effects for very high total IL-18 and CXCL9 levels in separate cohorts containing Stills and HLH patients. Though we did not observe a “hook effect” for CXCL9 or IL-18BP in an aptamer-based assay, the scarcity of very high CXCL9 or IL-18BP levels in that cohort precludes making any generalizations. These observations suggest that “proteomic” multi-analyte panels carry significant Type II error, and investigators should be cautious making any “failure to detect” conclusions from such data without proper controls.

Nevertheless, the biomarker discovery screen identified a few immunologic themes of potential importance to our understanding of IHF immunopathology. First, biomarkers of T-cell activation and IFNγ-activity were present broadly in IHF, including hyperferritinemic sepsis, and were not unique to one diagnosis (Fig. 5B, PC1). By contrast, Lin et al. showed that IFNγ-induced chemokines (CXCL9, CXCL10, CXCL11) differentiated HLH from sepsis and SIRS patients, although ferritin may have been equally effective in this all-comers sepsis/SIRS cohort(26). That biomarkers of T-cell activation appear equally upregulated in hyperferritinemic sepsis as in more classical causes of HLH adds to other lines of evidence suggesting shared pathogenesis(27–30). Additionally, we identified an unexpected separation (Fig. 5B, **PC2**) of hyperferritinemic sepsis samples from healthy controls and other IHF groups. This separation was driven by lower abundance of proteins like ANGPT1 and VEGFR2, and increased IL-15. One Olink analysis of a small adult sepsis cohort (ferritin status unknown) also showed lower VEGFR2 and higher IL-15 in sepsis patients compared to healthy controls(31) (**Supplemental Fig. 7**). Angpt-1 has been previously shown to be decreased in children with septic shock compared to critically ill children with either systemic inflammatory response syndrome (SIRS) or sepsis(31). Overall, the utility of Angiopoietin family members as biomarkers useful in subcategorizing sepsis has been studied without definitive conclusions, but never in comparable hyperinflammatory cohorts(32–35). If validated, these analytes could potentially play a future role in distinguishing hyperferritenemic septic patients from other IHF causes, such as HLH or MAS, aiding in early diagnosis and therapeutic management strategies. Notably, our dataset and analysis were unable to distinguish potential correlations of hypotension (or use of vasopressor medications) with biomarker values. Likewise, these analytes were not separately measured and we cannot rule out potential hook effects. Future studies should validate these findings and evaluate their clinical utility.

Ultimately, we implemented a single-center hyperferritinemic screening system and found it to be feasible and (both clinically and academically) useful. The data generated from this study confirmed patterns distinct to certain underlying etiologies, especially those associated of IL-18 with Stills. It also highlighted a potentially major short-coming, “hook effects” at high levels of specific analytes in highly multiplexed biomarker discovery platforms. Finally, our data also identified some potential areas of further exploration, such as depression of vascular growth factors in sepsis. Thus, leveraging the sensitivity of hyperferritinemia for HLH and related disorders via an alert system may be a window to improved recognition, management, and differentiation of this nebulous and life-threatening group of disorders.
